# Supplementary material for: Identification of photoperiod-induced specific miRNAs in the adrenal glands of Sunite sheep (Ovis aries)
Source: Front Vet Sci. 2022 Jul 22;9:888207. doi: 10.3389/fvets.2022.888207 (PMC9354845; doi:10.3389/fvets.2022.888207)
Supplement: Supplementary file 1 [file Data_Sheet_1.ZIP › Supplementary file/miRDeep2 file.zip!/miRDeep2 file/Know_Structure/SP42A1/oar-mir-379.pdf]

miRBase precursor : oar-mir-379
Total read count : 25223
oar-miR-379-5p read count : 23352
oar-miR-379-3p read count : 1866
remaining reads : 5

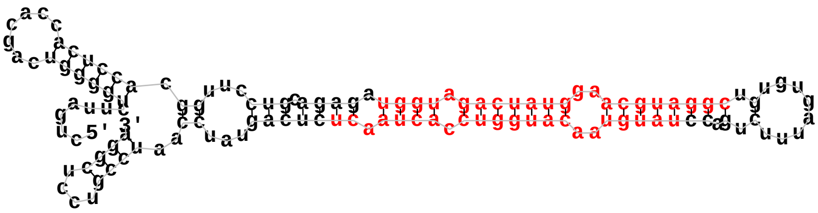

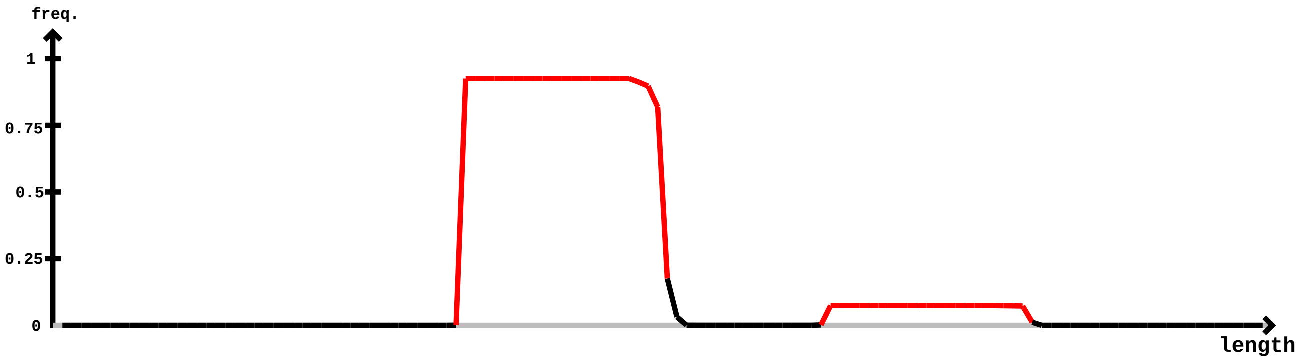


```
                                                                               oar-miR-379-3p
                                         oar-miR-379-5p
5'- cugauucuggggucagcaccacuccacgguuccugcagagaugguagacuauggaacguaggcugugugauuucugaccuauguaacaugguccacuaacucucaguauccaauccguccucgga  -3'    exp
    .......((((((.......)))))).((...(((.((((.((((.(((((((..((((((((.(........).).)))))))..))))))).)))).)))))))...))..((((....))))   reads     mm
    ........................................augguagacuauggaacguag................................................................   2         0
    ........................................augguagacuauggaacguagg...............................................................   11        0
    .........................................ugguagacuauggaacg...................................................................   2         0
    .........................................ugguagacuauggaacgu..................................................................   335       0
    .........................................ugguagacuauggaacgua.................................................................   387       0
    .........................................ugguagacuauggaacguag................................................................   1978      0
    .........................................ugguagacuauggaacguagg...............................................................   16206     0
    .........................................ugguagacuauggaacguaggc..............................................................   3630      0
    .........................................ugguagacuauggaacguaggcu.............................................................   782       0
    .........................................ugguagacuauggaacguaggcug............................................................   2         0
    .........................................ugguagacuauggaacguaggcugu...........................................................   3         0
    ..........................................gguagacuauggaacguag................................................................   1         0
    ..........................................gguagacuauggaacguagg...............................................................   8         0
    ..........................................gguagacuauggaacguaggc..............................................................   4         0
    ..........................................gguagacuauggaacguaggcu.............................................................   1         0
    ..............................................................cugugugauuucugacc..............................................   3         0
    ..............................................................cugugugauuucugacU..............................................   2         1
    ..............................................................................cuauguaacaugguccacua...........................   2         0
    ..............................................................................cuauguaacaugguccacuaac.........................   27        0
    ..............................................................................cuauguaacaugguccacuaacu........................   5         0
    ...............................................................................uauguaacaugguccacu............................   7         0
    ...............................................................................uauguaacaugguccacC............................   3         1
    ...............................................................................uauguaacaugguccacua...........................   10        0
    ...............................................................................uauguaacaugguccacuaa..........................   17        0
    ...............................................................................uauguaacaugguccacuaac.........................   1509      0
    ...............................................................................uauguaacaugguccacuaacu........................   284       0
    .................................................................................uguaacaugguccacuaac.........................   2         0
```
